# Patient-Derived Functional Models for Prediction of Radiotherapy Response in Rectal Cancer: A Systematic Review and Exploratory HSROC Meta-Analysis

**DOI:** 10.3390/life16071205

**Published:** 2026-07-21

**Authors:** Stefan Morarasu, Sorinel Lunca, Andrei-Nicolae Ceobanu, Alexandru-Florin Braniste, Gabriel Mihail Dimofte

**Affiliations:** 1 Grigore T Popa University of Medicine and Pharmacy, 700115 Iasi, Romania; morarasu.stefan@gmail.com (S.M.); mihail.dimofte@umfiasi.ro (G.M.D.); 2 2nd Department of Surgical Oncology, Regional Institute of Oncology, 700483 Iasi, Romania; 3 Department of Medical Oncology, Regional Institute of Oncology, 700483 Iasi, Romania

**Keywords:** rectal cancer, radiotherapy, xenograft, organoids, precision oncology, personalised medicine

## Abstract

**Background:** Patient-derived functional models have emerged as promising translational platforms capable of reproducing tumour-specific treatment sensitivity patterns, which could be used to personalise neoadjuvant treatment for patients with rectal cancer. Herein, we aimed to summarise the current comparative evidence in a meta-analytical framework on radiotherapy response between preclinical platforms and matched patient data. **Methods:** A systematic review was performed according to PRISMA principles to identify studies evaluating patient-derived functional models for the prediction of radiotherapy or chemoradiotherapy response in rectal cancer. Study characteristics, experimental protocols, predictive performance and clinical correlations were extracted. An exploratory hierarchical summary receiver operating characteristic (HSROC) meta-analysis was performed using studies providing sufficient data. **Results:** Eight studies involving patient-derived organoids and zebrafish patient-derived xenograft models were included. Most studies evaluated locally advanced rectal cancer treated with neoadjuvant chemoradiotherapy. The included studies demonstrated concordance rates ranging from 78% to 100% between ex vivo functional responses and matched clinical treatment outcomes. Reported predictive performance was favourable, with Yao et al. demonstrating 85.0% concordance, 78.0% sensitivity and 92.0% specificity, while Hsu et al. reported 87.5% sensitivity and 100% specificity using radiobiological modelling. Exploratory HSROC analysis demonstrated overall favourable discriminatory performance for prediction of treatment resistance and poor response. **Conclusions:** Patient-derived functional models, particularly PDOs, demonstrate promising potential as predictive biomarkers for radiotherapy and chemoradiotherapy response in rectal cancer. Although the current evidence remains exploratory and is limited by methodological heterogeneity and small cohorts, these platforms represent a promising translational strategy in precision radiation oncology, warranting prospective multicentre validation.

## 1. Introduction

Neoadjuvant therapy in the form of long-course/short-course chemoradiotherapy or total neoadjuvant therapy has dramatically improved outcomes in rectal cancer. Radiotherapy has reshaped the way we manage rectal cancer, first by enabling better local control and tumour downstaging, translating into unprecedented low rates of local recurrence. Secondly, in tandem with neoadjuvant chemotherapy, total neoadjuvant therapy (TNT) has led to a marked increase in complete responders and an ever-increasing adoption of the organ preservation strategy, commonly known as “watch and wait”, in up to one in five patients [1,2,3,4,5]. However, this paradigm shift has also highlighted an important limitation: response to radiotherapy is highly heterogeneous. A considerable proportion of patients do not show regression, having intrinsic resistance to radiotherapy, exposing patients to pelvic irradiation and its associated acute and long-term toxicities without proportional therapeutic benefit [6,7,8].

This variability in treatment response has important clinical implications. Patients achieving complete or near-complete response may benefit from organ-preserving strategies, while non-responders are exposed to treatment-related toxicity, delays to definitive surgery and potentially ineffective therapy without oncologic benefit. At present, our ability to accurately predict individual response to radiotherapy remains limited. Like other areas of medicine, we continue to rely largely on universal treatment paradigms, highlighting the need to transition toward a more personalised and precision-based approach to care. Predicting response to radiotherapy represents an important area of preclinical and translational research. Although several genomic, transcriptomic and immunologic signatures have been proposed, most fail to capture the dynamic and multifactorial nature of radiation sensitivity in vivo [9,10,11]. Consequently, there is a clear need for clinically relevant predictive platforms capable of reproducing the tumour microenvironment.

In recent years, patient-derived functional models have emerged as promising tools within the field of precision oncology [12,13]. These platforms include patient-derived organoids (PDOs), patient-derived xenografts (PDXs) and other avatar-based systems capable of preserving important biological characteristics of the original tumour. Compared with conventional two-dimensional cell cultures, patient-derived models better reproduce tumour heterogeneity, cellular architecture and treatment response patterns observed in clinical practice. Such models allow direct exposure of patient-derived tumour tissue to radiotherapy or chemoradiotherapy regimens, thereby enabling individualised assessment of radiosensitivity [14,15]. Several translational studies have suggested that responses observed in these ex vivo or in vivo models may correlate with actual clinical outcomes in matched patients with rectal cancer [16,17,18,19,20,21,22,23].

Among the available platforms, PDOs have attracted particular interest because of their relatively rapid establishment time, scalability and potential integration into real-time therapeutic decision-making [14,15,17,23]. Similarly, avatar-based approaches such as zebrafish xenografts have emerged as highly innovative strategies capable of providing rapid functional response assessment within clinically relevant timeframes compared with other animal models (i.e., murine) [24,25,26]. Nevertheless, despite increasing enthusiasm surrounding these technologies, the available evidence remains limited, being characterised by small cohorts with inconsistent reporting of predictive performance metrics and a lack of comparison with matched patient cohorts, thus eliminating their immediate translational potential. So far, no systematic synthesis has specifically evaluated the predictive accuracy of patient-derived functional models for radiotherapy response in rectal cancer using matched clinical outcomes.

Herein, we aim to perform a systematic review of the current evidence regarding patient-derived functional models as predictive platforms for radiotherapy response in rectal cancer. In addition, an exploratory diagnostic accuracy meta-analysis using hierarchical summary receiver operating characteristic (HSROC) modelling was performed in studies reporting sufficient matched patient-level response data.

## 2. Materials and Methods

### 2.1. Literature Search

**Figure 1:**
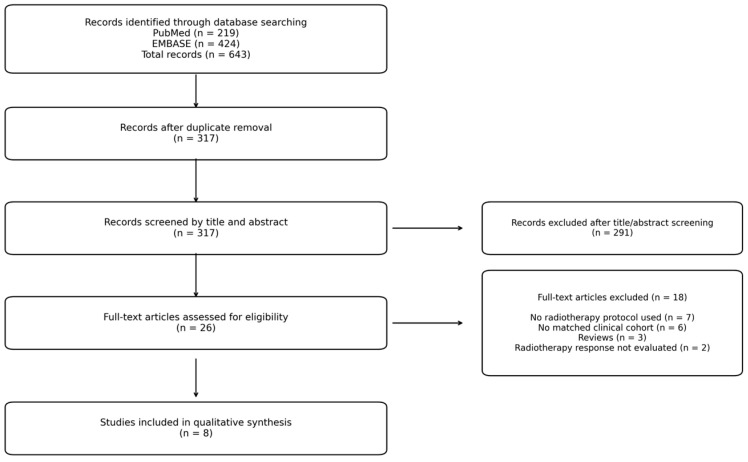
PRISMA flowchart.

This systematic review and exploratory diagnostic accuracy meta-analysis was registered with PROSPERO (International Prospective Register of Systematic Reviews) [27]. As PROSPERO does not yet support the registration of diagnostic accuracy meta-analysis, the study was registered under the closest applicable systematic review framework, while the diagnostic accuracy methodology and planned HSROC analyses were explicitly detailed within the protocol. The study ID is CRD420261385568. The Preferred Reporting Items for Systematic Reviews and Meta-Analyses (PRISMA) guidelines [28] were used as the search protocol, and the PRISMA checklist was followed to conduct the methodology (Figure 1). A systematic search of PubMed and EMBASE databases was performed from database inception to 20 April 2026 to identify studies evaluating patient-derived functional tumour models exposed to radiotherapy or chemoradiotherapy and correlated with matched patient clinical outcomes in colorectal or rectal cancer. The following search algorithm was used: (colorectal OR rectal OR colon) AND (patient-derived xenograft OR organoid) AND (radiotherapy OR radiation). The search strategy also included free-text keywords related to colorectal cancer, rectal cancer, radiotherapy, chemoradiotherapy, patient-derived organoids, patient-derived xenografts, zebrafish avatars and functional precision oncology platforms. The complete search strategies for each database are provided in Supplementary Table S1. In addition, the reference lists of eligible articles and relevant reviews were hand-searched to identify further studies.

### 2.2. Eligibility Criteria

Studies were considered eligible if they fulfilled the following criteria: (1) included patients with rectal cancer treated with radiotherapy or chemoradiotherapy; (2) evaluated a patient-derived functional model, including patient-derived organoids, patient-derived xenografts, zebrafish avatars or related patient-derived functional platforms; (3) exposed the model to radiotherapy and/or chemoradiotherapy ex vivo or in vivo; and (4) compared model-derived responses with matched patient clinical, radiological or pathological treatment outcomes.

Both prospective and retrospective translational studies were eligible for inclusion. Studies reporting sufficient data to evaluate concordance between model response and patient response were considered for qualitative synthesis. Studies reporting extractable data allowing the calculation of sensitivity and specificity were additionally considered for quantitative diagnostic accuracy synthesis.

**Figure 2:**
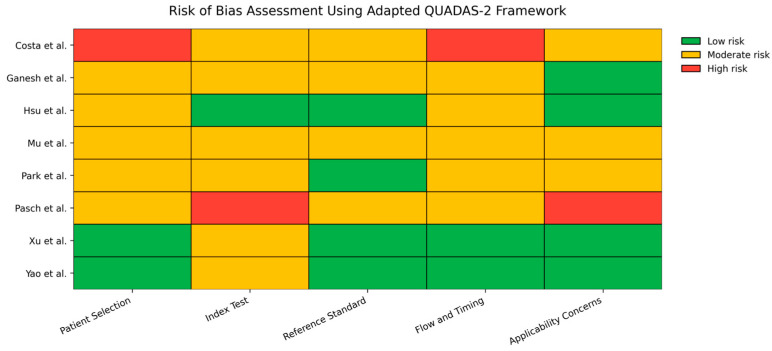
Visual overview of the risk of bias assessment adapted to the QUADAS-2 framework.

Exclusion criteria included studies using exclusively immortalised cell lines or non-patient-derived spheroids, studies lacking matched clinical patient outcomes, purely mechanistic radiobiology experiments without translational correlation, conference abstracts without sufficient methodological data, review articles, editorials and non-English publications. Two authors (SM and SL) assessed the titles and abstracts of studies found in the search, and the full texts of potentially eligible trials were reviewed. Disagreements were resolved by consensus-based discussion. Methodological quality and risk of bias were assessed using an adapted Quality Assessment of Diagnostic Accuracy Studies-2 (QUADAS-2) framework [29] (Figure 2, Supplementary Table S2). The four standard QUADAS-2 domains (patient selection, index test, reference standard, and flow and timing) were retained without modification. However, their interpretation was tailored to the translational setting. Specifically, the index test domain evaluated organoid or patient-derived xenograft establishment procedures, culture conditions, irradiation protocols, and whether functional response assessment was performed without knowledge of the corresponding clinical outcome. The reference standard domain assessed the appropriateness and consistency of clinical response definitions (e.g., pathological complete response, tumour regression grade, clinical complete response, or radiological response). The flow and timing domain considered whether patient-derived models were generated from pretreatment biopsies and whether all enrolled patients were included in the final correlation analysis, accounting for potential attrition due to unsuccessful model establishment or incomplete clinical follow-up. The corresponding author was contacted to clarify data extraction if additional information was necessary.

### 2.3. Data Extraction and Outcomes

For each eligible study the following data were recorded: study design, country, patient number, model establishment success rate, treatment modality, radiotherapy dose, chemotherapy regimen, timing of response assessment, pathological response criteria, clinical response definitions, and reported predictive metrics, including sensitivity, specificity, accuracy, and area under the curve (AUC). For studies eligible for quantitative diagnostic accuracy synthesis, contingency table data, including true positives (TP), false positives (FP), false negatives (FN) and true negatives (TN), were extracted directly or reconstructed from published response data and reported diagnostic performance metrics where feasible. For studies eligible for quantitative diagnostic accuracy synthesis, contingency table data (true positives, false positives, false negatives, and true negatives) were extracted directly whenever reported. When raw contingency tables were unavailable, but sufficient diagnostic performance metrics were provided, approximate 2 × 2 contingency matrices were reconstructed. Directly reported contingency data were available for Yao et al., whereas contingency tables for Hsu et al. and Park et al. were reconstructed from published diagnostic performance metrics and patient-level outcome data, respectively. The reconstructed datasets and reconstruction methodology are provided in Supplementary Table S3. The primary outcome of the review was the predictive concordance between patient-derived functional model response and matched patient clinical response to radiotherapy or chemoradiotherapy. Secondary outcomes included sensitivity, specificity and diagnostic accuracy.

### 2.4. Statistical Analysis

An exploratory diagnostic accuracy meta-analysis was performed for studies reporting sufficient data to construct contingency tables. Because of the limited number of eligible studies, small sample sizes and heterogeneity in response definitions and experimental methodologies, all quantitative analyses were considered exploratory. Sensitivity and specificity estimates were synthesised using a hierarchical summary receiver operating characteristic (HSROC) framework and bivariate random-effects modelling where feasible. Forest plots of sensitivity and specificity and HSROC curves were generated to evaluate overall predictive performance. Studies lacking sufficient contingency data for diagnostic synthesis were analysed descriptively, focusing on experimental protocols, radiotherapy protocols, predictive performance and clinical correlation.

## 3. Results

### 3.1. Eligible Studies

Eight studies [16,17,18,19,20,21,22,23] containing data on patient-derived preclinical models for the prediction of radiotherapy or chemoradiotherapy response in rectal cancer were included (Table 1). The initial search found 643 studies. After excluding duplicates and unrelated studies based on abstract triage, 26 full texts were assessed for eligibility, of which eight met the inclusion criteria and were systematically reviewed (Figure 1). The years of publication of included studies ranged from 2019 to 2025. The total number of included patients in the matched cohort for the preclinical models was 223. Seven studies used organoids, while one study used zebrafish xenografts [16] as the preclinical model. Three studies provided sufficient diagnostic performance data to allow reconstruction of contingency tables and inclusion in an exploratory diagnostic accuracy meta-analysis [18,20,23]. The remaining five studies were included in the narrative synthesis because of insufficiently extractable data, heterogeneous methodologies or exploratory study design.

### 3.2. Design of Patient-Derived Functional Models

Seven studies [17,18,19,20,21,22,23] adopted patient-derived organoids (PDOs) as experimental models while one study used patient-derived xenografts (PDXs) in zebrafish to correlate with clinical response [16]. In both cases, models were established from pretreatment endoscopic biopsies or surgical specimens obtained from patients with locally advanced rectal cancer undergoing neoadjuvant chemoradiotherapy. PDO establishment protocols were standardised across studies and involved enzymatic or mechanical dissociation of tumour tissue followed by 3D culture within extracellular matrix scaffolds such as Matrigel under stem cell-enriched culture conditions. Although all studies employed patient-derived organoid platforms, important methodological heterogeneity existed regarding tissue acquisition, organoid establishment techniques, culture conditions and integration with complementary in vivo validation models. An overview of protocols is detailed in Table 2.

### 3.3. Radiotherapy Protocols

Experimental treatment protocols varied considerably between studies. Most investigations exposed PDOs to external beam irradiation either alone or combined with fluoropyrimidine-based chemotherapy. Commonly evaluated agents included 5-fluorouracil, irinotecan or SN38, oxaliplatin, and combination regimens designed to increase the response rate.

Several studies assessed radiation response using viability assays following single-dose or fractionated irradiation protocols, while others incorporated more advanced radiobiological methodologies, including clonogenic survival modelling, D0 calculation, or organoid growth kinetics. More recent studies additionally integrated computational and machine learning approaches to assess predictive performance and response classification [20].

Besides their ability to predict radiotherapy response, PDOs were found to adequately identify responses to individual chemotherapeutic agents within multimodal treatments. Multiple studies demonstrated that organoids resistant to irradiation could remain sensitive to systemic agents, potentially explaining favourable patient responses despite intrinsic radioresistance. This observation suggests that PDOs may provide functional information capable of predicting the relative contribution of each component of multimodal neoadjuvant treatment (Table 3).

### 3.4. Predictive Performance

Clinical response assessment was heterogeneous across studies and included tumour regression grade (TRG), pathological complete response (pCR), clinical complete response (cCR), MRI-based response assessment, progression-free survival and composite responder/non-responder classifications. Despite methodological variability, most studies demonstrated favourable concordance between experimental model response and matched patient outcomes. Reported predictive performance metrics were generally high, with several studies reporting accuracy, sensitivity and specificity values approaching or exceeding 80–90% (Table 4).

### 3.5. Diagnostic Accuracy Meta-Analysis

Three studies [18,20,23] provided sufficient data for inclusion in the exploratory hierarchical summary receiver operating characteristic (HSROC) analysis. Across included studies, patient-derived organoid platforms demonstrated favourable predictive performance for identifying response to neoadjuvant radiotherapy or chemoradiotherapy. Individual study sensitivity ranged from approximately 78% to 93%, while specificity ranged from approximately 89% to 100%.

**Figure 3:**
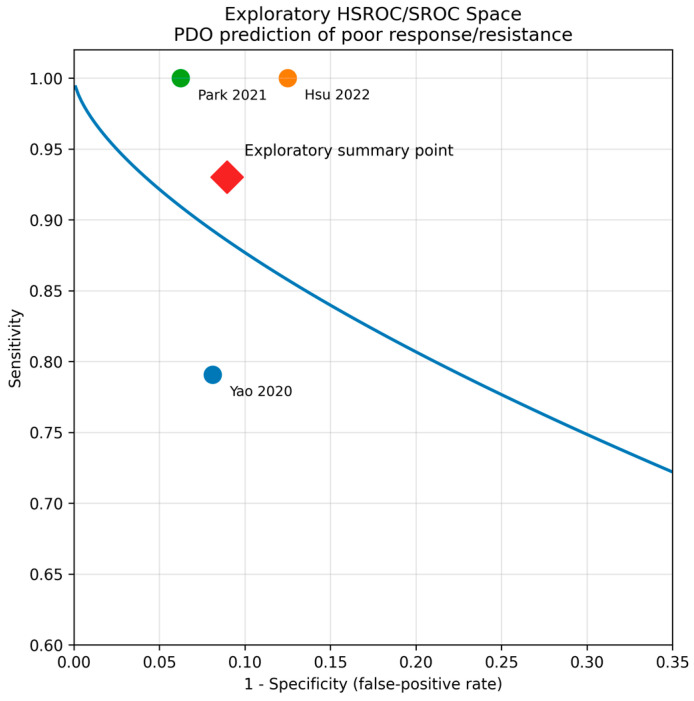
HSROC plot of the exploratory diagnostic accuracy meta-analysis evaluating patient-derived organoid (PDO)-based prediction of resistance or poor response to neoadjuvant radiotherapy/chemoradiotherapy in rectal cancer. Individual study estimates are displayed within ROC space according to sensitivity and the false positive rate (1—specificity). The red diamond represents the exploratory pooled summary estimate.

The exploratory HSROC analysis suggested favourable discriminatory performance; however, interpretation is limited by the inclusion of only three studies, heterogeneous response definitions, and reconstructed diagnostic datasets. Consequently, pooled diagnostic accuracy estimates should be considered hypothesis-generating rather than definitive. Approximate pooled performance metrics suggested sensitivity and specificity approaching 90%, with an estimated HSROC area under the curve between 0.92 and 0.95 (Figure 3, Supplementary Table S3). Study-specific sensitivity and specificity estimates are presented in Supplementary Figure S1.

## 4. Discussion

The present systematic review and exploratory diagnostic accuracy meta-analysis synthesised the currently available evidence regarding patient-derived functional models for the prediction of radiotherapy and chemoradiotherapy response in rectal cancer. Overall, the available evidence demonstrated consistent biologic concordance between experimental model response and matched patient outcomes, supporting the potential role of patient-derived functional platforms as predictive biomarkers for individualised radiotherapy response.

To our knowledge, this is the first meta-analysis to compare radiotherapy response between PDOs/PDXs and matched patient data, thus highlighting the translational potential of preclinical platforms. Our analysis highlights the translational potential of patient-derived functional models as predictive biomarkers for radiotherapy and chemoradiotherapy response while identifying the key methodological and clinical challenges that must be addressed before their routine implementation in precision oncology. Multiple studies demonstrated that organoid sensitivity patterns closely mirrored patient response following neoadjuvant chemoradiotherapy, with reported predictive performance frequently exceeding conventional expectations for translational biomarkers. Yao et al. [23] reported an overall concordance rate of 85.0%, with a sensitivity of 78.0% and a specificity of 92.0%, while Hsu et al. [18] demonstrated excellent discriminatory performance using radiobiological D0 modelling. Similarly, Park et al. [20] showed high predictive discrimination using machine learning-assisted PDO analysis. While the included studies were primarily designed as proof-of-concept investigations, the availability of matched patient cohorts strengthens the translational relevance and reliability of their findings. The exploratory HSROC analysis further supported the overall predictive potential of PDO-based models. However, interpretation of the exploratory HSROC findings requires caution. Clinical response assessment varied substantially across studies and included tumour regression grade (TRG), pathological complete response (pCR), clinical complete response (cCR), MRI-based assessment, and composite responder/non-responder classifications. Furthermore, differences in irradiation protocols, concurrent systemic therapies, and experimental methodologies introduce important sources of heterogeneity. Consequently, the HSROC analysis should be viewed as hypothesis-generating rather than a definitive pooled estimate of diagnostic accuracy.

From a translational perspective, the ability of PDOs to predict treatment resistance may become a cornerstone in rectal cancer management. Current neoadjuvant chemoradiotherapy strategies expose many patients to significant toxicity despite heterogeneous treatment benefit. Functional prediction platforms capable of identifying radioresistant tumours before treatment initiation could potentially guide alternative systemic strategies, selective intensification protocols or earlier surgical intervention. Similarly, highly radiosensitive tumours identified through PDO testing may promote the use of boosted radiotherapy protocols to maximise the chance of complete responses and organ preservation.

Although patient-derived organoids demonstrate considerable promise as predictive biomarkers, several practical challenges must be overcome before routine clinical implementation. Establishment success rates and assay turnaround times were inconsistently reported across studies, making it impossible to determine whether organoid-based testing can reliably fit within the narrow window preceding neoadjuvant radiotherapy or chemoradiotherapy. Despite this, the zebrafish patient-derived xenograft (zPDX) model described by Costa et al. demonstrated rapid engraftment and functional assessment within a clinically relevant timeframe, suggesting that alternative patient-derived avatar models may complement or even replace PDOs when rapid treatment decisions are required. Nevertheless, this approach remains preliminary and requires validation in larger prospective cohorts. In addition, substantial variability in biopsy processing, culture conditions, extracellular matrix systems, irradiation protocols, and response assessment currently limits reproducibility and cross-study comparability. Future prospective multicentre studies should therefore incorporate standardised methodologies while systematically reporting establishment success, assay turnaround time, and workflow feasibility to facilitate translation into routine clinical practice.

Patient-derived functional models should also be considered within the broader landscape of emerging predictive biomarkers used to guide the management of colorectal cancer. Predicting response to neoadjuvant therapy is currently one of the most active areas of investigation in precision oncology for locally advanced rectal cancer. Recent advances in circulating tumour DNA (ctDNA), MRI-based radiomics, and deep learning algorithms have shown considerable promise for predicting treatment response and identifying patients suitable for organ-preserving strategies [30,31,32,33,34]. These approaches offer important practical advantages, including their non-invasive nature, scalability, and potential for repeated longitudinal assessment. However, unlike imaging- or blood-based biomarkers, patient-derived organoids provide a direct functional assessment of individual tumour sensitivity to radiotherapy and chemotherapeutic agents, potentially capturing complex biological interactions within the tumour microenvironment that may not be reflected by molecular or imaging signatures alone. Compared with blood-based biomarkers, PDOs require tumour tissue acquisition, specialised laboratory infrastructure, longer assay turnaround times, and currently lack standardised culture protocols. So far, the results favouring the use of PDOs as avatars for personalised rectal cancer treatment originate from small, pilot/experimental studies without robust patient group comparisons, while blood biomarkers, especially ctDNA, are supported by large prospective randomised trials [34]. Ultimately, these technologies should not be viewed as competing approaches but rather as complementary strategies, with the integration of functional testing, liquid biopsy, and advanced imaging offering a more comprehensive precision oncology framework for individualised treatment selection.

Several limitations must be acknowledged. First, substantial heterogeneity existed regarding organoid establishment techniques, extracellular matrix systems, irradiation protocols, chemotherapy combinations, response thresholds and clinical outcome definitions. This variability limited direct comparability between studies. In addition, the included investigations used a variety of endpoints to define treatment response, including tumour regression grade (TRG), pathological complete response (pCR), clinical complete response (cCR), MRI-based response assessment, and composite responder/non-responder classifications. While all endpoints aimed to capture sensitivity to radiotherapy or chemoradiotherapy, they reflect different biological and clinical dimensions of treatment response and are not fully interchangeable. Second, most available studies were single-centre translational investigations with relatively small patient cohorts, increasing the risk of selection bias and limiting external generalisability. Third, only a minority of studies reported complete contingency data necessary for a proper diagnostic accuracy meta-analysis, emphasising the need for future standardised reporting of outcomes. Another limitation relates to the intrinsic complexity of modelling radiotherapy response ex vivo. Although PDOs preserve important tumour-intrinsic characteristics, they incompletely reproduce several clinically relevant components of the tumour microenvironment, including stromal interactions, immune infiltration, vascularisation and systemic treatment effects. An additional source of variability relates to differences in extracellular matrix scaffolds used for organoid culture, including Matrigel and basement membrane extract. Variations in matrix composition, biomechanical properties, and growth factor content may influence organoid proliferation, viability, and cellular responses to radiation, further highlighting the need for standardised culture protocols in future studies. Another major unresolved issue, which created confusion on the feasibility of using such models in clinical practice, is whether patient-derived functional testing can be completed within the standard interval between diagnostic biopsy and initiation of neoadjuvant therapy. Although this appears feasible in selected studies, the time required for PDO establishment and functional testing was not systematically reported across the available literature, precluding determination of a reliable average turnaround time. Standardised reporting of assay turnaround time and establishment success should therefore be considered essential endpoints in future prospective validation studies. In addition, only studies published in English were included, which may have introduced language bias by excluding potentially relevant evidence published in other languages.

Despite these limitations, the present study highlights several strengths of patient-derived functional modelling, reflecting the need for more translational studies that may accelerate the clinical implementation of personalised neoadjuvant therapy in patients with rectal cancer based on the ex vivo response of PDOs/PDXs. Future validation should be performed through prospective multicentre studies enrolling consecutive patients with locally advanced rectal cancer treated with standard neoadjuvant therapy. Pretreatment biopsies should be collected from all patients, patient-derived functional models should be established using standardised protocols, and functional responses should be assessed in a laboratory blinded to clinical outcomes. The predictive performance of these models should then be prospectively compared with predefined clinical outcomes, including pathological complete response, regression grade (Dworak grading), and clinical complete response. Such studies would provide more robust evidence regarding the reproducibility and clinical utility of patient-derived functional models.

## 5. Conclusions

Patient-derived functional models, particularly patient-derived organoids, show promising potential as predictive biomarkers for radiotherapy and chemoradiotherapy response in locally advanced rectal cancer. The available evidence demonstrates encouraging concordance between ex vivo functional responses and matched clinical outcomes, suggesting that these models may contribute to future precision oncology strategies. However, given the limited number of eligible studies, methodological heterogeneity, and the exploratory nature of the quantitative synthesis, the findings of the HSROC meta-analysis should be interpreted with caution and considered hypothesis-generating rather than definitive estimates of diagnostic accuracy. Further prospective multicentre studies using standardised methodologies and uniform reporting of diagnostic performance are required to validate these models before routine clinical implementation.

## Figures and Tables

**Table 1:** Overview of studies.

Study	Model Type	Patient Cohort	Treatment Evaluated
Costa 2020 [16]	PDX	2 patients	CRT
Ganesh 2019 [17]	PDO	Translational cohort	CRT
Hsu 2022 [18]	PDO	13 patients	CRT
Mu 2025 [19]	PDO ± PDX	19 patients	CRT
Park 2021 [20]	PDO	33 patients	CRT
Pasch 2019 [21]	PDO	Translational cohort	Radiotherapy
Xu 2025 [22]	PDO	Translational cohort	CRT
Yao 2020 [23]	PDO	80 patients	CRT

**Key:** PDO, patient-derived organoid; PDX, patient-derived xenograft; CRT, chemoradiotherapy.

**Table 2:** PDO/PDX experimental protocol overview.

Study	Tissue Source	PDO Establishment Method	Culture Matrix/Medium
Costa 2020 [16]	Rectal tumour tissue	Mechanical and enzymatic dissociation followed by zebrafish xenografting	Not applicable
Ganesh 2019 [17]	Primary and metastatic rectal cancer tissue	Tumour dissociation and embedding into 3D extracellular matrix cultures	Basement membrane extract/matrigel with organoid-supportive medium
Hsu 2022 [18]	Rectal cancer endoscopic biopsies	Enzymatic digestion and organoid propagation under stem cell conditions	Matrigel-based colorectal organoid medium
Mu 2025 [19]	Rectal tumour tissue	Standardised 3D PDO establishment after tissue dissociation	Extracellular matrix-supported stem cell medium
Park 2021 [20]	Rectal cancer biopsy specimens	Tumour fragmentation and enzymatic dissociation into PDO cultures	Matrigel-based organoid medium
Pasch 2019 [21]	Rectal cancer surgical specimens	PDO establishment from patient tumours using 3D culture conditions	Basement membrane matrix with colorectal organoid medium
Xu 2025 [22]	Locally advanced rectal cancer biopsies	Large-scale PDO biobank generation and propagation	Matrigel-based organoid expansion medium
Yao 2020 [23]	Rectal cancer endoscopic biopsies	Mechanical and enzymatic tissue dissociation followed by PDO establishment	Matrigel and colorectal organoid stem cell medium

**Key:** PDO, patient-derived organoid.

**Table 3:** Overview of radiotherapy and chemoradiotherapy protocols.

Study	Model	Experimental Treatment Protocol	Main Outcome Assessed
Costa 2020 [16]	PDX	Single-dose irradiation (25 Gy)	Tumour apoptosis and concordance with patient response
Ganesh 2019 [17]	PDOs	Chemoradiotherapy exposure assays	Functional treatment sensitivity and response phenotypes
Hsu 2022 [18]	PDOs	Clonogenic radiation survival assays; D0 modelling	Radiosensitivity and clinical response correlation
Mu 2025 [19]	PDOs/PDOX	5-FU, irinotecan, and oxaliplatin ± 8 Gy irradiation	PDO response, apoptosis and pathologic response
Park 2021 [20]	PDOs	Experimental irradiation with a machine learning prediction model	Prediction of responder versus non-responder status
Pasch 2019 [21]	PDOs	Radiation and chemotherapy sensitivity assays	Metabolic and growth-based treatment response
Xu 2025 [22]	PDOs	8 Gy irradiation combined with 5-FU ± irinotecan	Organoid response kinetics and pTRG/cCR prediction
Yao 2020 [23]	PDOs	Irradiation (0–16 Gy) combined with 5-FU and/or CPT-11	TRG/cCR prediction and treatment response concordance

**Key:** PDO, patient-derived organoid; PDX, patient-derived xenograft; TRG, tumour regression grade; cCR, clinical complete response.

**Table 4:** Predictive performance and clinical correlation of patient-derived functional models.

Study	Predictive Performance	Clinical Correlation
Costa 2020 [16]	Concordant radiosensitivity phenotypes observed in zebrafish PDX models	Experimental response correlated with matched patient CRT response
Ganesh 2019 [17]	PDOs identified heterogeneous treatment sensitivity patterns	Organoid response reflected patient-specific CRT response
Hsu 2022 [18]	Sensitivity 87.5%; specificity 100%	PDO radiosensitivity strongly correlated with clinical response
Mu 2025 [19]	PDO/PDOX models reproduced sensitive and resistant phenotypes	Experimental response paralleled pathological outcomes
Park 2021 [20]	Exploratory sensitivity 100%; specificity 93.8%	PDO radiosensitivity correlated with pathological response
Pasch 2019 [21]	Heterogeneous treatment susceptibility identified by metabolic imaging	Functional response signatures reflected treatment sensitivity
Xu 2025 [22]	High concordance between PDO response and patient outcome	Organoid kinetics correlated with CRT response
Yao 2020 [23]	Concordance 85.0%; AUC 88.2%; sensitivity 78.0%; specificity 92.0%	PDO profiles closely matched TRG and cCR outcomes

**Key:** PDO, patient-derived organoid; PDX, patient-derived xenograft; TRG, tumour regression grade; cCR, clinical complete response; CRT, chemoradiotherapy; AUC, area under the curve.

## Data Availability

No new data were created or analysed in this study. Data sharing is not applicable to this article.

## Supplementary Materials

The following supporting information can be downloaded at https://www.mdpi.com/article/10.3390/life16071205/s1 Supplementary Table S1: Search Strategy; Supplementary Table S2: Risk of bias assessment; Supplementary Table S3: Contingency table used for HSROC analysis; Supplementary Figure S1: Sensitivity and Specificity Forest Plots.

## Abbreviations

The following abbreviations are used in this manuscript:

AUC	Area Under the Curve
cCR	Clinical Complete Response
CRT	Chemoradiotherapy
EMBASE	Excerpta Medica Database
FN	False Negative
FP	False Positive
HSROC	Hierarchical Summary Receiver Operating Characteristic
MRI	Magnetic Resonance Imaging
pCR	Pathological Complete Response
PDO	Patient-Derived Organoid
PDOX	Patient-Derived Organoid Xenograft
PDX	Patient-Derived Xenograft
PRISMA	Preferred Reporting Items for Systematic Reviews and Meta-Analyses
QUADAS-2	Quality Assessment of Diagnostic Accuracy Studies-2
ROC	Receiver Operating Characteristic
TNT	Total Neoadjuvant Therapy
TN	True Negative
TP	True Positive
TRG	Tumour Regression Grade
