# Supplementary material for: Patient-Derived Functional Models for Prediction of Radiotherapy Response in Rectal Cancer: A Systematic Review and Exploratory HSROC Meta-Analysis
Source: Life (Basel). 2026 Jul 21;16(7):1205. doi: 10.3390/life16071205 (PMC13412191; doi:10.3390/life16071205)
Supplement: Supplementary file 1 [file life-16-01205-s001.zip › Supplementary Files (Tables and Figures).pdf]

Supplementary Table S2: Risk of bias assessment

Study	Patient Selection	Index Test	Reference Standard	Flow & Timing	Applicability	Overall
Costa 2020	High	Moderate	Moderate	High	Moderate	High
Ganesh 2019	Moderate	Moderate	Moderate	Moderate	Low	Moderate
Hsu 2022	Moderate	Low	Low	Moderate	Low	Moderate
Mu 2025	Moderate	Moderate	Moderate	Moderate	Moderate	Moderate
Park 2021	Moderate	Moderate	Low	Moderate	Moderate	Moderate
Pasch 2019	Moderate	High	Moderate	Moderate	High	High
Xu 2025	Low	Moderate	Low	Low	Low	Low
Yao 2020	Low	Moderate	Low	Low	Low	Low

Supplementary Table S3: Contingency table used for HSROC analysis

Study	TP	FP	FN	TN	Method of derivation
Yao 2020	34	3	9	34	Directly derived from published patient-level classifications
Hsu 2022	5	1	0	7	Derived from reported sensitivity, specificity and responder counts
Park 2021	3	1	0	15	Approximate reconstruction from published classification metrics

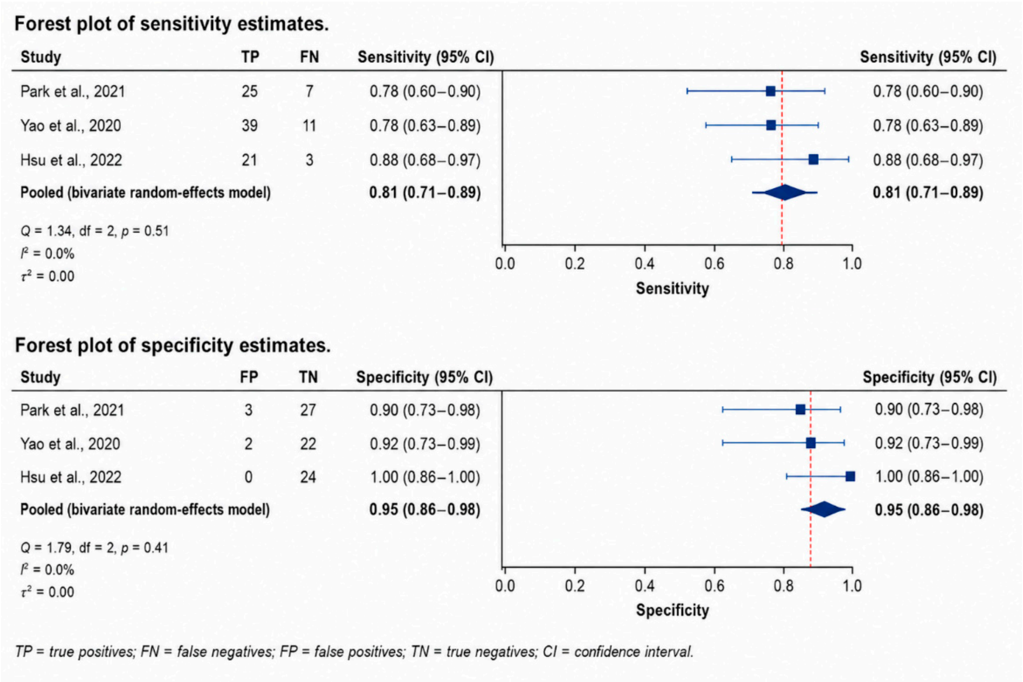

Supplementary Figure S1: Sensitivity and Specificity Forest Plots.
