# Supplementary material for: Patient-Derived Functional Models for Prediction of Radiotherapy Response in Rectal Cancer: A Systematic Review and Exploratory HSROC Meta-Analysis
Source: Life (Basel). 2026 Jul 21;16(7):1205. doi: 10.3390/life16071205 (PMC13412191; doi:10.3390/life16071205)
Supplement: Supplementary file 1 [file life-16-01205-s001.zip › Supplementary Table S1 - SEARCH STRATEGY.pdf]

| SEARCH CRITERIA | |
|---|---|
| **PUBMED SEARCH CRITERIA** | (("Colorectal Neoplasms"[Mesh] OR colorectal cancer OR rectal cancer OR colon cancer)) AND (("Xenograft Model Antitumor Assays"[Mesh] OR xenograft* OR "patient-derived xenograft*" OR PDX OR organoid* OR "patient-derived organoid*" OR PDO OR "3D culture" OR tumoroid*)) AND (("Radiotherapy"[Mesh] OR radiotherapy OR radiation OR irradiation OR radiosensitivity OR radioresistance)) AND ((patient* OR clinical OR "co-clinical" OR matched OR paired OR correlation OR concordance OR predictive OR "treatment outcome"[Mesh] OR "treatment response")) |
| **EMBASE SEARCH CRITERIA** | ('colorectal cancer'/exp OR 'rectal cancer' OR 'colon cancer') AND ('xenograft'/exp OR 'patient derived xenograft' OR pdx OR organoid* OR '3d culture' OR tumoroid*) AND ('radiotherapy'/exp OR radiotherapy OR radiation OR radiosensitivity OR radioresistance) AND (patient* OR clinical OR 'co-clinical' OR matched OR paired OR correlation OR concordance OR predictive) |
